# *sunflower*: an R package for handling multiple response attempts and conducting error analysis in aphasia and related disorders

**DOI:** 10.3389/fpsyg.2025.1538196

**Published:** 2025-02-14

**Authors:** Ismael Gutiérrez-Cordero, Javier García-Orza

**Affiliations:** ^1^Numerical Cognition Lab, Universidad de Málaga, Málaga, Spain; ^2^Department of Basic Psychology, Universidad de Málaga, Málaga, Spain; ^3^Cognitive Neurology and Aphasia Unit, Centro de Investigaciones Médico-Sanitarias (CIMES), Universidad de Málaga, Málaga, Spain; ^4^Instituto de Investigación Biomédica de Málaga (IBIMA), Málaga, Spain

**Keywords:** R package, Speech Therapy, language assessment, paraphasia classification, language production

## Abstract

Manual classification of production errors and the allocation of speech/spelling scores are time-consuming, laborious and error-prone tasks, even when conducted by clinicians and specialized researchers. Here we present *sunflower*, an R package developed to improve the analysis of language production quality for Spanish data. The package offers various functions, including (1) managing dataframes containing single responses and multiple-attempt responses, (2) conducting formal similarity analyses on words as well as positional accuracy data analyses within words, and (3) the classification of errors by considering lexicality, formal similarity and semantic similarity indexes, which are obtained by means of different algorithms and artificial intelligence techniques such as word2vec. The applications of *sunflower*, which is the first open-source package of its kind, include assessing whether production quality improves over the course of multiple attempts, and identifying which aspects of an individual’s productions are most impacted by their impairments. Other potential applications include the analysis of whether improvements arise in a patient’s production quality after a given treatment, distinguishing between cases of apraxia of speech and conduction aphasia, as well as simply using the package to improve and speed up the classification of speech/spelling errors with large datasets through automation.

## 1 Introduction

The analysis of errors in both spoken and written language is of considerable relevance in clinical and experimental contexts within the field of Speech Therapy and (Neuro)psychology of Language, from cross-sectional or experimental assessments (e.g., García-Orza et al., 2020; Gold and Kertesz, 2001; Goodglass and Wingfield, 1997; Gutiérrez-Cordero and García-Orza, submitted) to longitudinal, treatment-related ones in subjects with aphasia or related impairments (e.g., Berthier et al., 2018) and those with other speech-language disorders, such as stuttering and developmental language disorders (e.g., Einarsdóttir et al., 2024).

These analyses allow for the formulation of specific profiles of patients, rather than simply positioning them on a unidimensional scale, which is the usual procedure. They permit a more in-depth understanding of the state of individuals’ psycholinguistic mechanisms, and the study of errors thus allows us to develop not only better diagnostic or treatment tools, but also to contribute to discussions about models of language production (e.g., Dell et al., 1997; Dotan and Friedmann, 2015; Levelt et al., 1999).

To analyze errors, researchers and clinicians typically rely on manual transcriptions and subsequent error classifications (e.g., how to classify *zebra* as a response to the presentation of *giraffe*) or on the scoring of production performance (e.g., compute the degree to which *draft* and *giraffe* are similar). Such forms of analysis are widely seen as time-consuming, laborious, and prone to errors, even when carried out by clinicians (such as speech therapists and expert neuropsychologists) or specialist researchers (e.g., Themistocleous et al., 2021).

Furthermore, assessment requires an explicit comparison of the participant’s response to a target word, usually letter by letter (or phoneme by phoneme if spoken) (e.g., Caramazza and Miceli, 1990); to do so, it is also necessary to consider what kind of processes are being engaged in the case of misproduction (e.g., deletions, substitutions, simplification of consonant clusters, etc.). When assessment is manual, there is a great potential for errors to be made at this point, including oversights, changes in the criteria during the process, the coder’s assumptions or perception-based biases, and errors in the computations of metrics (e.g., Kent, 1996).

Formal errors serve as a good example of production errors. These are commonly produced by patients with lexical impairments, and are errors in which both the response (a real word) and the target word share at least 50% of the elements therein (phonemes or letters depending on the production modality) or simply share the start (initial phoneme/letter) (e.g., Gold and Kertesz, 2001; García-Orza et al., 2020; Goodglass and Kaplan, 1972). Thus, in the case of producing *nonsense* for *nuisance* the second criteria is met, and this is straightforward, but in the case of *intelligence* (as response) and *development* (as target) things become more complicated and an explicit test for the former criterion is needed. Here, one cannot rely on what is apparent to the naked eye (they are not short words like *pale* and *cale*), essentially because such an analysis is not reliable; instead, the proportion of shared letters (*psl*) should be computed, as follows:


$$psl=\left(\frac{SL\times 2}{NLt+NLr}\right)\times 100=\left(\frac{6\times 2}{11+12}\right)\times 100=52.17\%$$

where *SL* is the number of shared letters, *NLt* is the total number of letters in the target word, and *NLr* is the number of letters in the response. Also, 2 is a fixed value indicating that these letters [(3 times) *e, l, n, t*] are present in both words. In this case the computation has focused on the orthographic transcription, but clearer results would be returned with phonemic (broad) transcriptions [/dɪˈvɛl.əp.mənt/ (*development*) and /nˈtɛl.ɪ.dʒəns/ (*intelligence*)] in addressing the phonological form of these two strings, for which the proportion of shared phonemes (*psp*) would be 63.64% (after removing stress and syllable separation marks).

The examples just given are relatively simple, but classifying errors can become a somewhat more complicated task. This is the case, for example, with semantic errors (e.g., giraffe for hippo) and even more so with mixed errors [e.g., /ˈraɪnoʊ/ (*rhino*) for /ˈhɪpoʊ/ (*hippo*)]; in the latter, the clinician/researcher might erroneously categorize the error as semantic, even when it also “formally” meets the criteria for a formal error (*psl* = 60% and *psp* = 54.55%) by overlooking the target–response formal similarity (example from Nelson et al., 2020).

Turning to the formal analysis of production quality in research studies and clinical practice (either by comparing patients’ samples, tasks or pre−/post-treatment assessments), measures such as the *psl* and *psp* described above would continue to be insufficient in that they do not offer much information about the productions per se. In these cases, other indexes, such as the longest common subsequence (*lcs*) of two strings [e.g., for *mangrove* (target) and *mango* (response), *lcs* = mang], the number and proportion of correct characters in their corresponding position [the hits for *mang*(*rove*) are 11110000, 50%], and edit distances, such as Damerau–Levenshtein’s [3 (deletion processes in *r*, *v* and *e*) in this case] (e.g., Smith et al., 2019), might shed more light on the quality and nature of these productions. Such formal indexes can, however, be rather difficult, or indeed impossible, to compute by hand, but they can be obtained automatically. Relying on this kind of metric, more frequently seen in the area of genetics (e.g., Berger et al., 2020), makes possible a more exhaustive and reliable assessment of spoken and written production (Gutiérrez-Cordero and García-Orza, in preparation; Haley et al., 2023; Smith et al., 2019; Themistocleous et al., 2021), offering considerable advantages over standard practices that extend to both experimental and translational research.

Another facet of studies on production errors is the analysis of repeated attempts. Sometimes an individual produces more than a single response when presented with a stimulus. Normally, the first complete production is the one considered (e.g., Laganaro, 2005), but there are occasions in which these verbal repetitive behaviors are of special interest (e.g., Joanette et al., 1980). This is the case, for example, with people who stutter, those with apraxia of speech, and those with conduction aphasia; such individuals usually offer more than a single response in the form of repetitive attempts (RA) (e.g., Ramoo et al., 2021; Gutiérrez-Cordero and García-Orza, in preparation). Focusing on the latter type of individuals, who show phonological impairments, it is common to find frequent instances of *conduite d’approche* (CdA), a kind of repetitive verbal behavior involving successive self-corrective attempts as they try to reach a given word (e.g., “*unirve*, *inuv*, *imurno*, *unives*, *universe*” for *universe*) (Torres-Prioris et al., 2019). Handling and managing dataframes in which multiple responses are provided to some stimuli is potentially difficult, although this is a minor issue, one that can easily be addressed by being consistent in collecting and registering responses transcriptions in a dataset. It is worth noting here that over more than 30 years, the study of repeated attempts such as CdAs has received very little attention, this probably due at least in part to the practical difficulties in addressing the issue (e.g., Joanette et al., 1980; Marshall and Tompkins, 1982; Valdois et al., 1989).

When it comes to categorizing the kind of response produced by a participant or patient by deciding whether a given response entails a real word or a nonword, and also considering both the formal and semantic similarities of such response regarding the target word, the task becomes yet more problematic. The best approach seems to be to automate these lexicality and formal checks for similarity by means of algorithms and to consider the relationship of the response with the target words by using AI models (e.g., Salem et al., 2023a; Schnur and Lei, 2022; see also Azevedo et al., 2024, for a review), after which—naturally—the outputs should be supervised. To the best of our knowledge, there are currently two AI-based studies that focus on the classification of errors in single word production assessment in aphasia. Le et al. (2017) used automatic speech recognition technology to discriminate only between phonemic and neologistic errors, whereas Fergadiotis et al. (2016) developed a highly accurate tool that was able to categorize formal, semantic, mixed, neologistic and unrelated errors.

In the present study we present *sunflower*, a tool that allows for the categorization of responses and errors. The development of this tool in the form of an R package is motivated by the need to automate and speed up the categorization of verbal/written responses as much as possible while also enabling further computations related to the formal analysis of speech/spelling performance. This is the first work to provide a freely accessible tool for this purpose, and is designed specifically for the Spanish language. We hope that this package will help clinicians and researchers in their work with dataframes, allowing them to manage and deal with large amounts of data in more time-efficient ways, computing complex measures, and improving the consistency and quality of their practices, all with the final goal of conducting finer and more revealing analyses on the resulting data.

## 2 Methods

### 2.1 R implementation and dependencies

The *sunflower* package was developed in the *R* programming language (version 4.2.2—“Innocent and Trusting”; R Core Team, 2022)^1^ using the *RStudio* IDE (version 4da58325, 2024-01-28—“Ocean Storm”; RStudio Team, 2024)^2^, which was chosen due to its widespread use in our field, thus ensuring reproducible research.

The dependencies of *sunflower* are the *tidyverse* (Wickham, 2023), whose core comprises packages such as *dplyr* (Wickham et al., 2022), *purrr* (Wickham and Henry, 2023), *stringr* (Wickham, 2022), *tibble* (Müller and Wickham, 2023), and *tidyr* (Wickham et al., 2024a,b), as well as *magrittr* (Bache and Wickham, 2022), *PTXQC*, *reshape2* (Wickham, 2007), *rlang* (Henry and Wickham, 2024), *stringdist* (van der Loo, 2014), *tictoc* (Izrailev, 2023) and *word2vec* (Wijffels et al., 2023).

### 2.2 Repository access and availability

The *sunflower* R package has been made available in an active repository on GitHub,^3^ but can also be accessed in an associated OSF mirror repository.^4^ In this mirror, we provide some *additional files to be downloaded*, such as in the case of the word2vec model allocated in the dependency-bundle zip file, essentially because these are required in order to take full advantage of all the functions we provide with *sunflower*, like those in the Step 3: Classify Errors section.

This package is licensed under the GNU General Public License version 3 (GPLv3), see the LICENSE file in the root directory of the package for more details.

### 2.3 Functions provided by the package

The *sunflower* R package is developed to assist with three main tasks, working stepwise:

**Step 1:** Managing and wrangling data provided by any individual involving multiple items and responses, as well as supporting work with transcriptions (either orthographic or phonemic) previously done regarding responses entailing any number of attempts (as occurs in instances of CdA).

**Step 2:** Computing various measures of formal similarity and other related indexes, which are difficult to compute manually, if not impossible (e.g., Haley et al., 2023; Themistocleous et al., 2021), as well as providing a fine-grained assessment of the positional accuracy of assessed material.

**Step 3:** Conducting a psycholinguistic classification of errors that relies on an initial check as to whether the responses produced are real words or not (lexicality check), and sorting responses in terms of both formal and semantic similarity measures following classical criteria established in the field (e.g., Dell et al., 1997).

**Figure 1:**
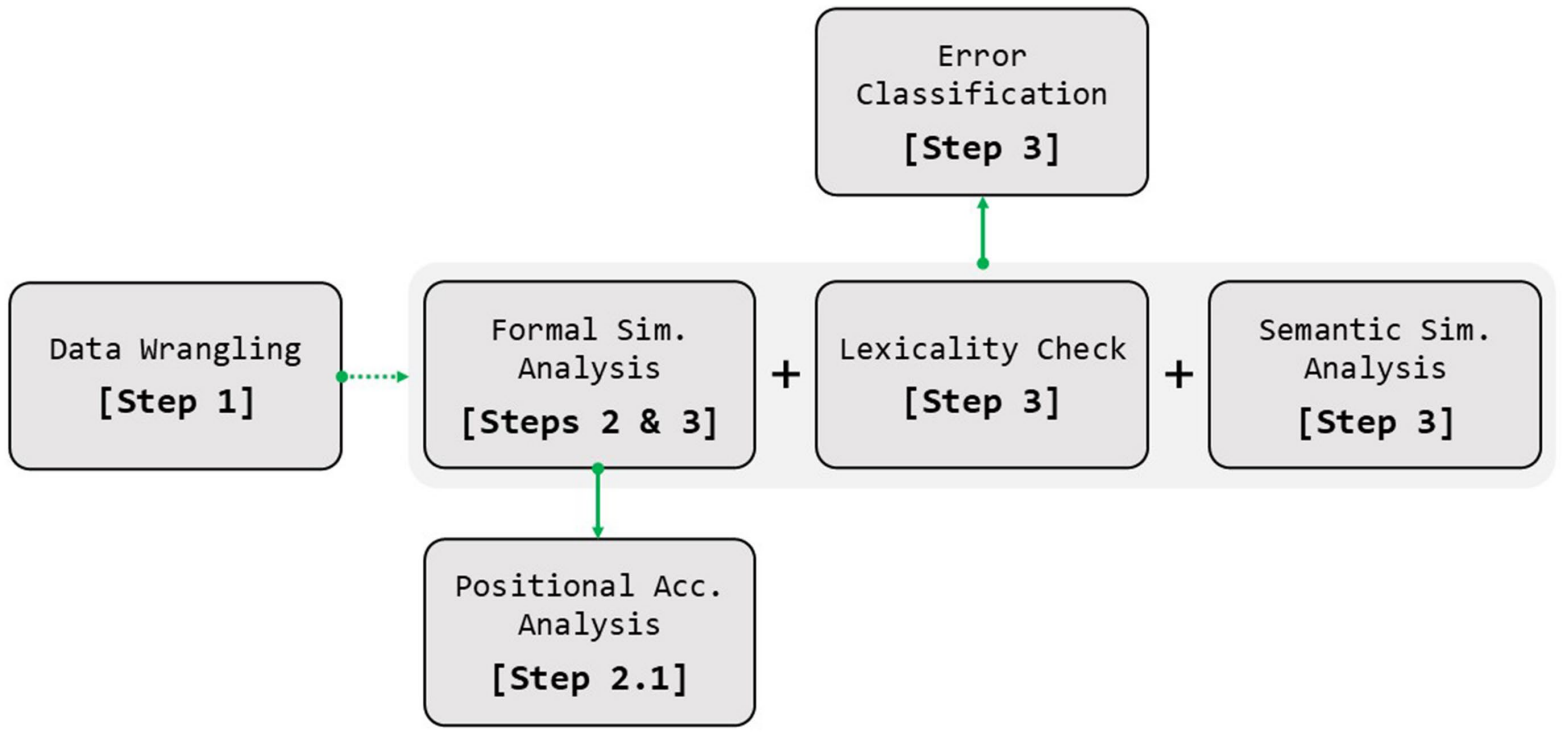
Steps followed to apply the main functions of the *sunflower* package. Sim., similarity; Acc., accuracy.

In the following sections, we describe how to install the package and take advantage of its functionalities, along with code examples to address the abovementioned tasks and the steps followed to achieve them. These steps are also represented in the diagram in Figure 1.

## 3 Installation

The *sunflower* R package can be installed using the following command in R: devtools::install_github(“ismaelgutier/sunflower”). The user should make sure that they have the *devtools* package (Wickham et al., 2022) installed on their machine in order to be able to install *sunflower* from the GitHub repository. If *devtools* is not installed, the user can do so using install.packages(“devtools”). Once the package is installed, it can be loaded to work with the command library(“sunflower”).

## 4 Working with the package

In this section, we present a working example based on the data collected by Gutiérrez-Cordero and García-Orza (in preparation) after administering a series of tasks. Specifically, the dataset we begin working with in this example is named IGC_sample. For clarity and ease of use, a subset of these data has been made available as several datasets, with their properties outlined in Table 1. The datasets labeled "long" in the table are the direct result of applying the functions described in Step 1: Manage Repetitive Attempts to IGC_sample. Additionally, the dataset with "phon" in its name contains the phonemic (broad) transcriptions of both the items and the responses, these obtained by means of the procedure detailed in footnote 7. Simulated data (simulated_sample) are also included to allow users to test the functions presented in subsequent sections.

**Table 1:** Description of the datasets made available in the package.

Dataframe name	Rows	Columns	Column names	Further description
IGC_sample	386	7	ID, task, item_ID, item, response, correct, accessed	A portion of the dataset collected by Gutiérrez-Cordero et al. (in preparation).
IGC_long_sample	681	8	ID, task, item_ID, item, response, accessed, RA, attempt	A portion of the dataset collected by Gutiérrez-Cordero et al. (in preparation) is presented in long format, equivalent to the output obtained after applying the functions described in Step 1 of the text.
IGC_long_phon_sample	681	10	ID, task, item_ID, item, response, item_phon, response_phon, accessed, RA, attempt	A portion of the dataset collected by Gutiérrez-Cordero et al. (in preparation), which includes columns containing characters in IPA notation, was obtained following the process described in Footnote 7.
simulated_sample	75	5	item_ID, item, response, task_name, assessment_date	

**Figure 2:**
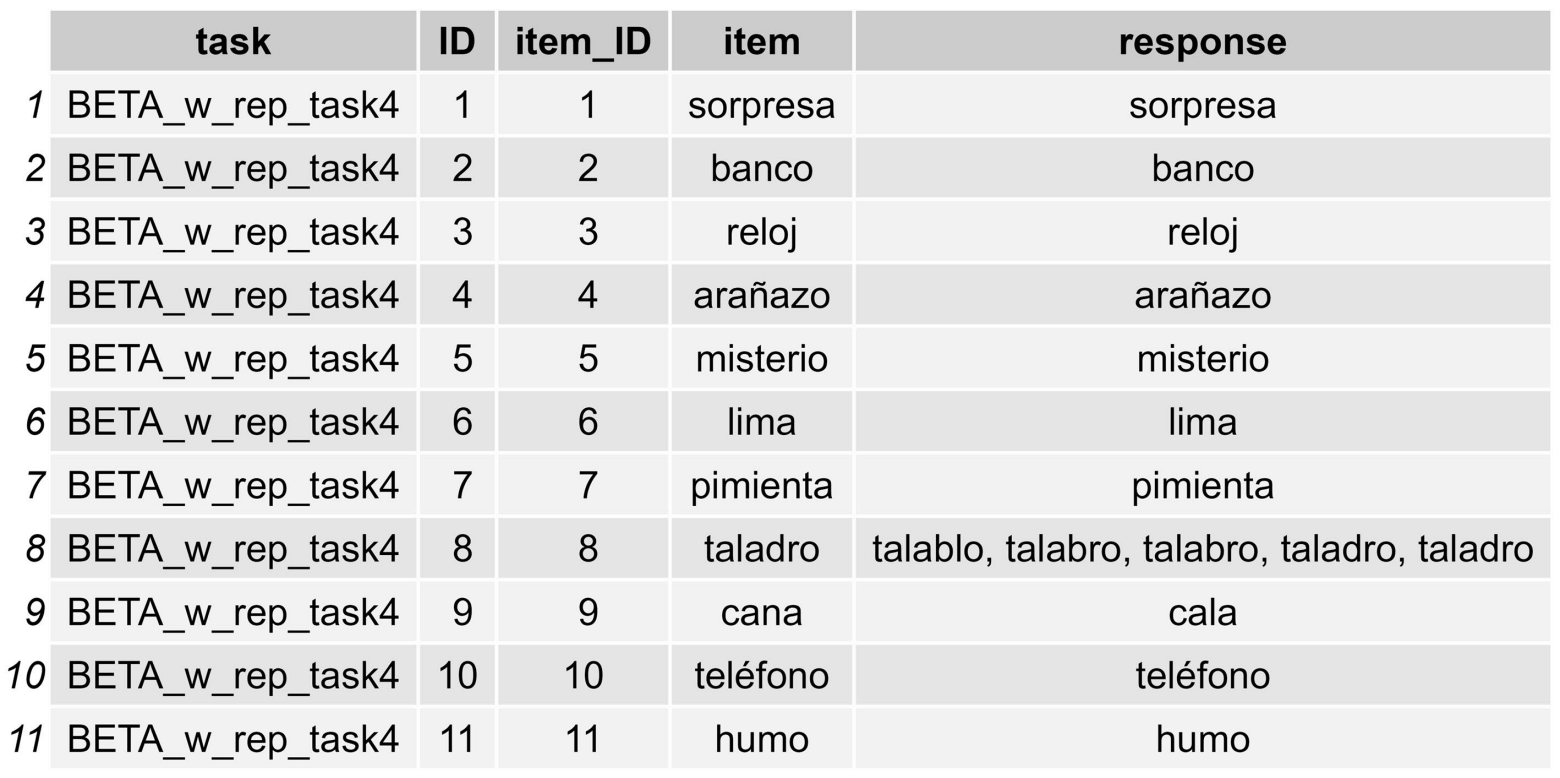
Initial working dataframe with instances of multiple responses for some items.

Users can load any of these datasets using the following lines of code, the idea being to work with a dataframe similar to the one shown in Figure 2.

(1)
#df with attempts together
     IGC_sample = sunflower::IGC_sample
#df with separated attempts (after Step 1) and phonemic transcriptions
     IGC_long_phon_sample = sunflower::IGC_long_phon_sample

Different datasets may be loaded from a personal Excel file or other file format. However, it is important that any initial dataframe must have the following kinds of data: item (e.g., *peine*), response (e.g., *pente, peine*), and a group of identifiers such as item ID (e.g., 1), task name (e.g., EPLA), or assessment date (e.g., 03–11), for example. We will now show how to apply the functions offered by *sunflower* to this initial dataframe.

### 4.1 Step 1: Manage repetitive attempts

**Figure 3:**
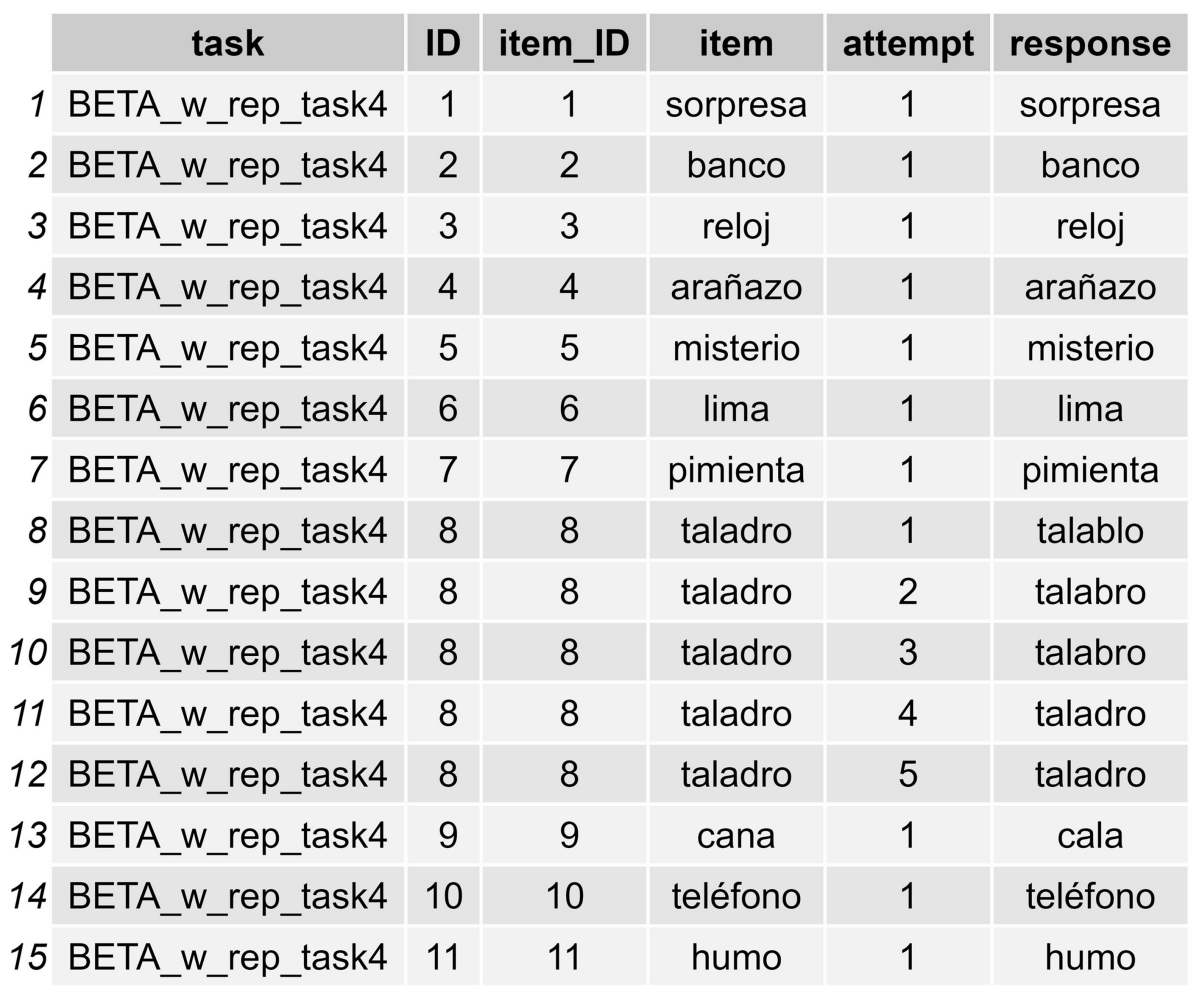
Long-format transformation of the same items from Figure 2, but with each response on a separate row. In this long-format dataframe it is indicated whether a RA has occurred, along with the attempt number within that RA.

In order to be able to apply the functions presented in what follows, the data must be structured in a specific way. That is, when the dataframe contains instances with multiple attempts in the response column, as occurs in the “response” column in the dataframe we are using here (IGC_sample) (see Figure 2, where there is a response with RAs, “*talablo*, *talabro, talabro, taladro, taladro*” for the item *taladro* [*drill* in Spanish]), this must be submitted by means of a data wrangling process which allows the user to split such attempts in the same response in different “instances.” The goal is to have each response displayed in separate cells across rows (a different row for each attempt provided for the word *taladro*). In other words, for any instance of RAs, each attempt in the response column must be displayed in a different row rather than within the same initial cell (we anticipate that they will appear as shown in Figure 3). The user might find it useful to have raw response transcriptions in a column, which can later be cleaned—by removing annotations or interjections, for example—so they can work with a column containing the clean responses in which only the pure intended productions are displayed, as occurs in Figure 3. Again, in those cases with RAs, the user needs to first separate the cleaned responses into different columns using the functions in the package, and then convert the data from wide format to long format. This process allows for the subsequent computation of formal metrics (Step 2: Compute Formal Similarity Measures), as well as for obtaining positional accuracy data (Step 2.1: Conduct Positional Accuracy Analysis), and the classification of errors (Step 3: Classify Errors) that enable both data visualization and statistical analyses.

To do so, we can proceed in a stepwise manner using a *tidyverse* style that relies on pipes,^5^ which makes the application of subsequent processes easy to understand. In the following code snippet, we first separate the responses of the initial dataframe into columns (as exemplified in Figure 2) and then directly convert these columns into rows, ending up with a long format (as is displayed in Figure 3) dataframe to work with.

(2)
df_long_format_w_attempts = IGC_sample %>%
               separate_responses(col_name = "response",
                    separate_with = ", ") %>%
               get_attempts(drop_blank_spaces = TRUE)

When applying this code to a dataframe, only two parameters need to be defined in the separate_responses() function: the col_name parameter, which is the column named “response” in our dataframe—it is important that the names of columns in our dataframe are written with quotation marks in the code; and for the separate_with parameter, which is the marker used to separate each attempt within the same item response [e.g., in our transcription of “*talablo*, *talabro, talabro, taladro, taladro*” for *taladro*, we used ”, ” (a comma followed by an empty space, which again needs to be defined between quotation marks in the code)].

The get_attempts() function does not need any parameters to be defined since it works directly with the output columns from the separate_responses() function; however, we recommend setting the parameter drop_blank_spaces = TRUE (or T) to remove any resulting empty rows being generated by the separate_responses() function, which will help to streamline the data, making it easier to analyze and report.

The code snippet above will return a new dataframe called df_long_format_w_attempts with three new columns: (1) a “RA” column indicating whether a repeated attempt (in our case a CdA) has been produced or not (repetition [1], single response [0]) for a given item; (2) another column called “attempt” which indicates how many instances these CdAs in the RA column entailed; and (3) a final column called “response” showing each single instance of response (see Figure 3).^6^

### 4.2 Step 2: Compute formal similarity measures

The study of the formal quality of productions in people with aphasia and related impairments has attracted considerable attention over the last few years. In this section we show how *sunflower* can be used to compute not only the indexes used in recent work here, such as the Damerau-Levenshtein distance (e.g., Themistocleous et al., 2021) or measures derived from it (Smith et al., 2019; Haley et al., 2023; Gutiérrez-Cordero and García-Orza, in preparation), but also some others mentioned above in the Introduction (such as the *lcs* or the proportion of phonemes/letters produced in the correct position).

The *sunflower* package not only allows for the possibility of working with the orthographic transcriptions of responses, but also—to a certain degree—with their phonemic (broad) transcriptions.^7^

At this point, a series of formal quality indexes, described in Tables 2, 3, can be computed using the responses registered in the df_long_format_w_attempts dataset, using the following code snippet:

(3)
df_w_formal_indexes = df_long_format_w_attempts %>%
     get_formal_similarity(
          item_col = "item",
          response_col = "response",
          attempt_col = "attempt",
          group_cols = c("ID", "item_ID"))

**Table 2:** Description of the formal similarity indexes obtained with the get_formal_similarity() function.

Variable	Description
targetL	Length of the target word string.
responseL	Length of the response string.
p_shared_char	The proportion of characters shared between the target and response strings. This ratio is calculated by dividing the number of shared characters (multiplied by two to account for their occurrence in both strings) by the total length of the target plus response strings.
p_shared_char_in_pos	The proportion of characters shared between the target and response strings in their correct position.
diff_char_num	The difference in character counts between the target and response strings.
Ld	The Levenshtein distance (Ld) measures the minimum number of single-character edits, specifically insertions, deletions, or substitutions, required to transform one word into another (is represented as an integer).
DLd	The Damerau–Levenshtein distance (DLd) is similar to the Levenshtein distance but also accounts for transpositions of adjacent characters, in addition to insertions, deletions, and substitutions, needed to transform one string into another.
JWd	The Jaro–Winkler distance (JWd) measures the edit distance between two strings based on matching characters and transpositions, producing a value between 0 (identical strings) and 1 (no similarity) (Winkler, 1990).
pcc	The proportion of correct phonemes (pcc) can be obtained as a measure of direct orthographic or phonemic performance by simply subtracting the result of dividing the DLd by the total number of phonemes from 1 (Gutiérrez-Cordero et al., in preparation; Haley et al., 2023).

**Table 3:** Description of the formal similarity indexes obtained with the get_formal_similarity() function (cont.)

Variable	Description
lcs	The Longest Common Subsequence is the longest subsequence that can be derived from two strings without changing the order of the characters.
similarity_str	Similarity vector between target and response strings, with values indicating: M (match), D (deletion), S (substitution), I (insertion).
shared1char	A Boolean value indicating whether the target and response strings start with the same character (TRUE) or not (FALSE).
strict_match_pos	A binary string that represents, for each position, whether the characters in the target and response strings match (where 1 = match and 0 = unmatch).
adj_strict_match_pos	Represents, like strict_match_pos, the character matches between the target and response strings, but is adjusted to the length of the target string.
comment_warning	Adds a warning if the response contains spaces or commas, which could indicate repeated attempts (RA) responses.
approach_diff	Measures the change (*Δ*) in the proportion of correct characters (pcc) between consecutive attempts within each group defined by group_cols. Useful for analyzing changes in performance across multiple attempts. This variable is directly dependent on how the attempt_col and group_cols parameters are defined.

Where the item_col and response_col parameters refer to the columns containing the items and the responses. As previously noted, we can use columns that contain either orthographic representations (e.g., *vaca* [*cow* in Spanish]) or phonemic transcriptions (/baka/). In our case, we used the orthographic transcriptions of both items and responses. These two columns are essentially the ones to be compared: “item” and “response,” respectively. We then need to specify two further parameters that will serve as pivotal identifiers for computing the formal similarity indexes: (1) the attempt_col parameter, which is set to NULL by default when repeated attempts (the RAs) are not involved, is defined as “attempt” in our case. Note that this “attempt” column is returned by the functions we applied in Step 1: Managing Repetitive Attempts, and it represents both the order and total count of attempts within the repeated attempts (RAs) that constitute the CdAs. And (2) the group_cols parameter, for which a group of columns (or at least one column) must be defined to serve as identifiers. In our case, we used two columns, which we refer to as the vector of variables: c(“ID,” “item_ID”), to ensure the correct organization of the data.

**Figure 4:**
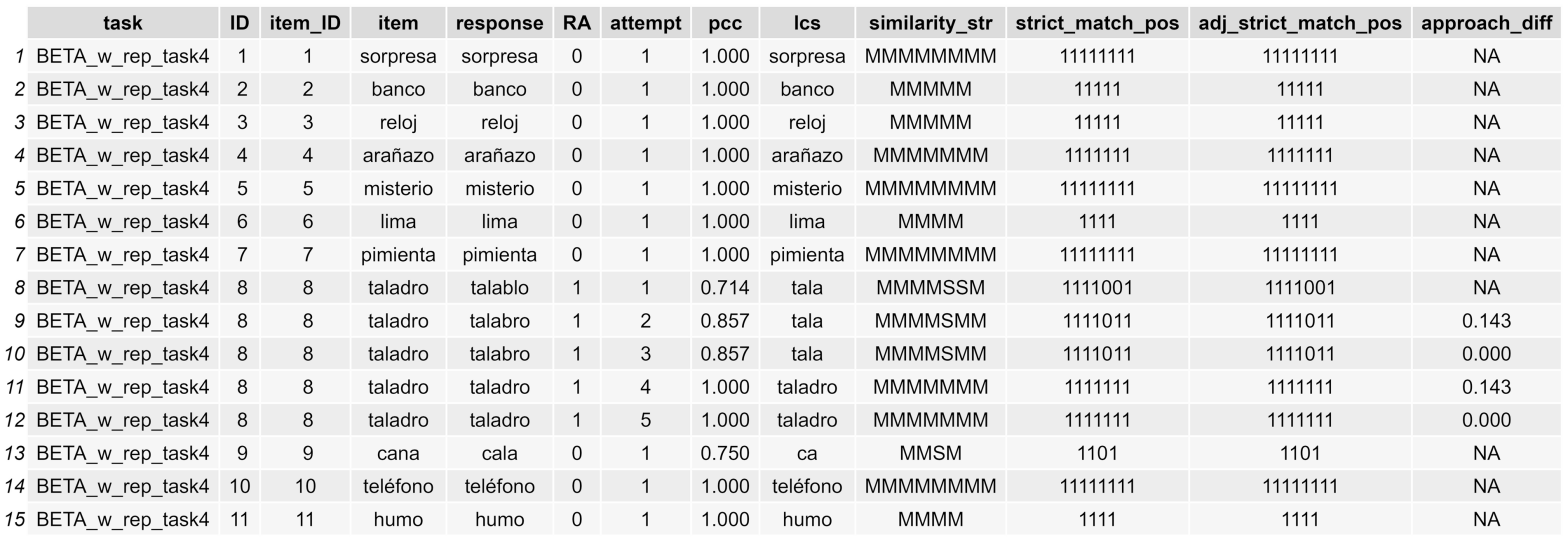
Long-format dataframe showing the formal indexes computed for orthographic transcriptions (cont.).

**Figure 5:**
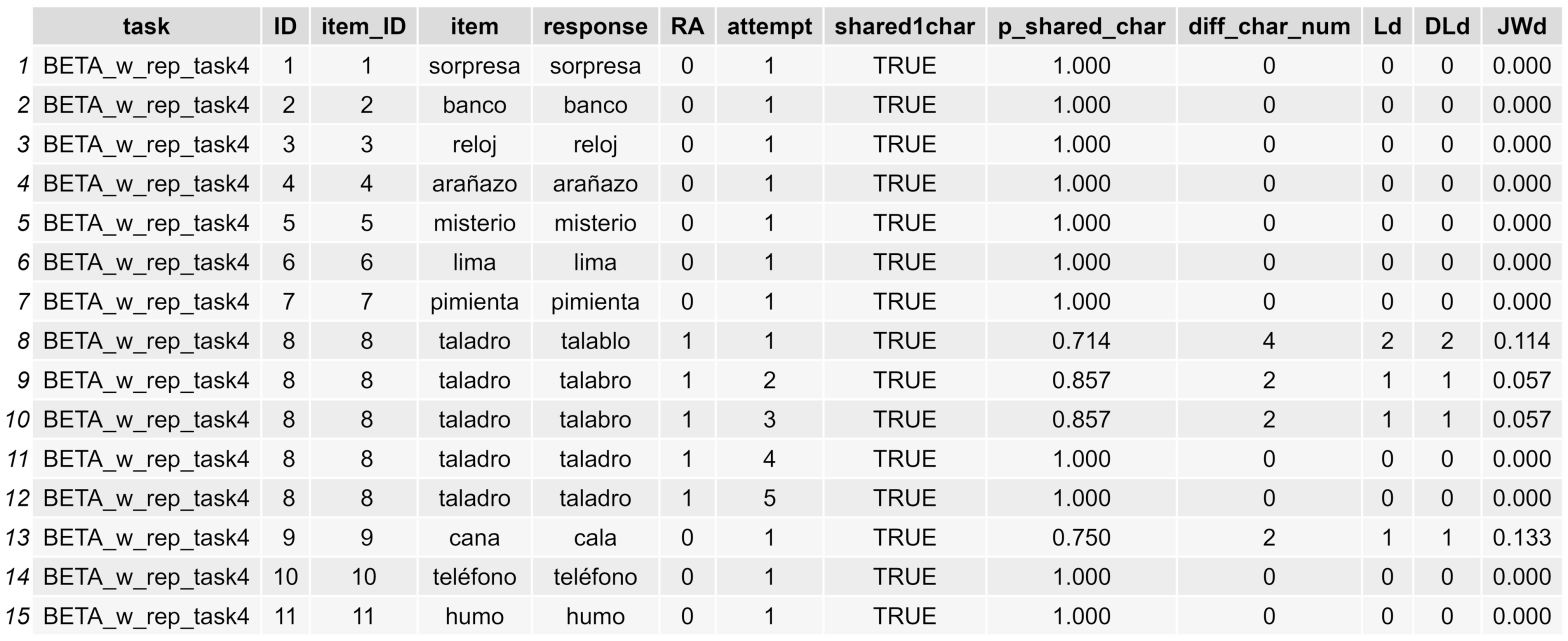
Long-format dataframe showing the formal indexes computed for orthographic transcriptions.

The logic underlying this approach is that we need to arrange the dataframe in such a way that we can compute the *approach_diff* index (see Figure 4). We must group by every possible confounding ID variable that could cause issues. For example, for the item *tornillo* in a specific task, we might want to analyze the evolution of the second attempt in relation to the first, and similarly for the third attempt in relation to the second. Thus, it is crucial to ensure that these attempts correspond to the same ID for this specific item within a particular task or assessment. If we were to overlook this, we might mistakenly compare the #2 attempt of a CdA produced during an assessment conducted in November with the #1 attempt of a CdA produced in an assessment conducted in August, whereas in fact we clearly want to compare the #2 and #1 attempt of the CdAs produced in each evaluation independently. If we set every parameter correctly, we will obtain a new df_w_formal_indexes with all the indexes described in Tables 2, 3, and shown in Figures 4, 5. If the parameters attempt_col and group_cols are omitted, the similarity measures are calculated without considering the *approach_diff* index.

Alternatively, we could work using phonemic (broad) transcriptions, in that *sunflower* supports working with IPA symbols (see above). Since the phonemic transcriptions are already stored in a separate dataframe in long format (i.e., IGC_long_phon_sample), we will need to load it and specify the relevant columns for comparison by setting the item_col and response_col parameters to “item_phon” and “response_phon” respectively. The remaining parameters (attempt_col and group_cols) would stay the same. The code snippet would be as follows, and the output would be named df_w_formal_indexes_phon.

(4)
df_w_formal_indexes_phon = sunflower::IGC_long_phon_sample %>%
     get_formal_similarity(
          item_col = "item_phon",
          response_col = "response_phon",
          attempt_col = "attempt",
          group_cols = c("ID", "item_ID"))

Thus far we have processed the initial dataframe and derived formal measures for the stimulus–response pairs. At this stage, as shown in Figure 1, the workflow diverges into two different paths. Users can either perform a positional analysis of the productions (Step 2.1) or continue with the steps required for a psycholinguistic classification of errors (Step 3).

### 4.3 Step 2.1: conduct positional accuracy analysis

The positional accuracy analysis provides information on the correct production of elements (either letters or phonemes). Positional accuracy data can be of great value in the study of speech errors in that it provides evidence for the underlying nature of the encoding impairments in syndromes such as apraxia of speech and conduction aphasia (Ramoo et al., 2021; Gutiérrez-Cordero and García-Orza, in preparation; Romani et al., 2002, 2011). This is particularly relevant when considering impairments at the phonemic level related to the phonological encoding (Dell, 1986; Dell et al., 1997; or more specifically, the phonological output buffer; García-Orza et al., 2020; Gutiérrez-Cordero and García-Orza, submitted) or processes which are not in themselves linguistic but are linked to articulatory processes, such as the phonetic encoding of articulatory programming (Levelt et al., 1999).

In Step 2: Compute Formal Similarity Measures, we showed how to obtain two strictly matching position indexes, one raw and the other adjusted to the target word’s length. We can use these matching strings to obtain the positional accuracy data of each response via the positional_accuracy() function, as shown in the following code lines:

(5)
df_positional_accuracy = df_w_formal_indexes %>%
     positional_accuracy(item_col = "item",
          response_col = "response",
     match_col = "adj_strict_match_pos")

In this code, the item_col parameter specifies the column containing the items for which we want to assess positional accuracy. In this case, they were the orthographic transcriptions of target words, whose column was called “item.” The response_col parameter refers to the column that contains the responses we are evaluating, which is the “response” column in our dataset. This same process could be applied to a dataframe containing phonemic transcriptions (e.g., df_w_formal_indexes_phon). It would only require adjusting the columns to item_phon and response_phon, with everything that follows remaining unchanged. The match_col parameter reads data from a column that contains the adjusted strict-matching-position strings, called “adj_strict_match_pos” that is obtained in the Step 2: Compute Formal Similarity Measures, specifically designed to reflect the positional accuracy of the responses relative to the items’ length (see Table 3 for a description).

**Figure 6:**
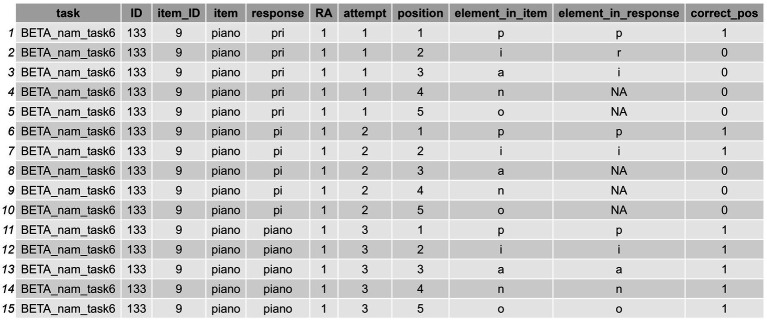
Long-format dataframe showing the positional accuracy data (characters produced correctly in their strict position per word and attempt) of a sample assessed.

**Figure 7:**
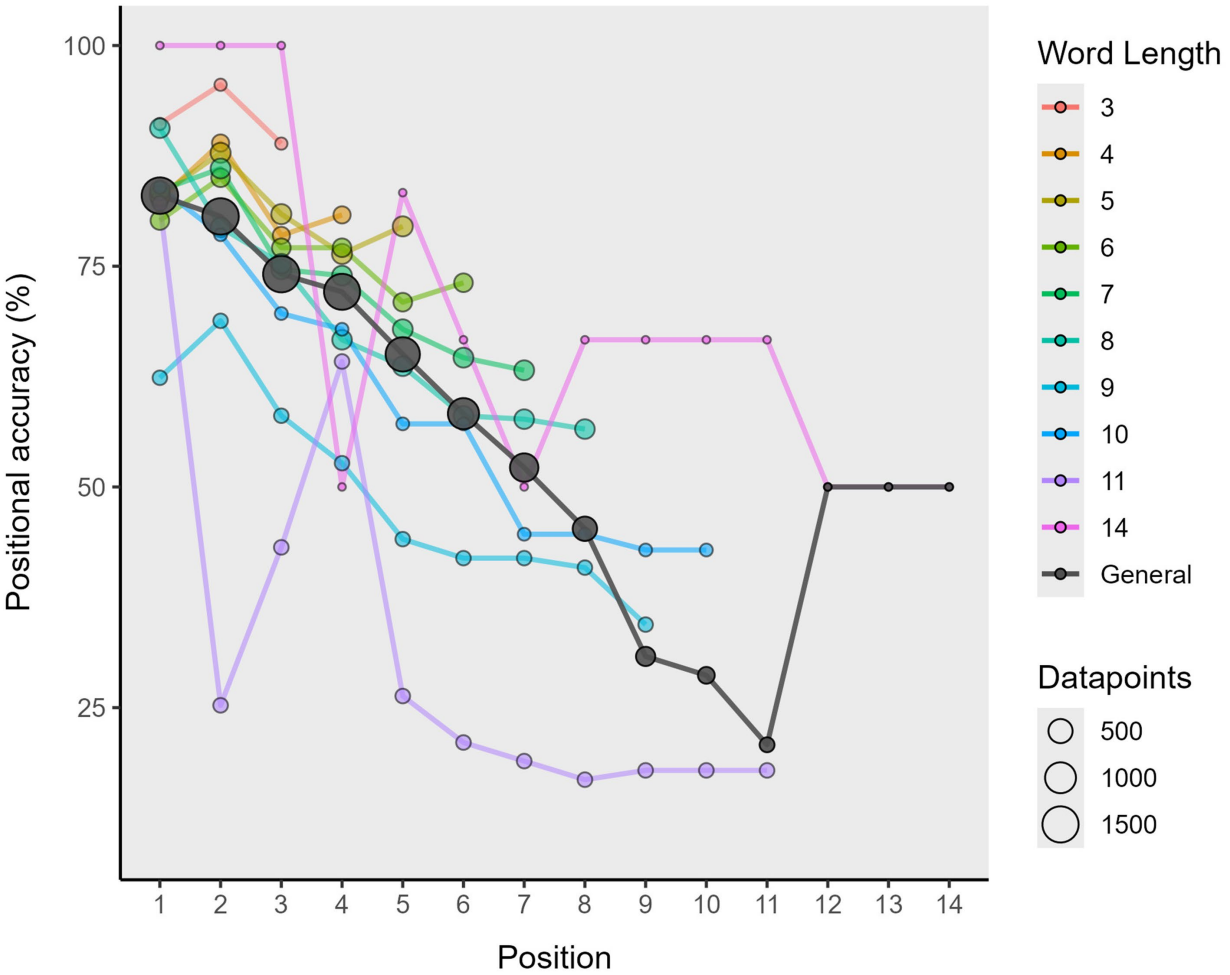
Graphical representation of the positional accuracy data of words assessed in different tests.

After applying this function, we obtain a more elongated df_positional_accuracy, with various new columns related to the positional accuracy analysis. Specifically, we obtain three new identifier columns, these called: (1) “position,” which indicates the position of the element addressed (either letter or phoneme) taking the item string (not the response) as a reference; (2) “element_in_item,” which provides the specific element in that position in the item; and (3) “element_in_response” that does the same for the element in the response string. However, an additional fourth column called “correct_pos” will also be provided, which indicates whether the element addressed in a given position within the target item is produced correctly or not. Likewise, other, prior identifiers for each word, such as the “attempt” column or “item_ID,” are retained. The output of this process is shown in Figure 6, and in a graphical representation in Figure 7.

### 4.4 Step 3: Classify errors

In this final section we focus on how to conduct the automatic classification of errors following the criteria of established typologies in the field (e.g., Dell et al., 1997; Gold and Kertesz, 2001; García-Orza et al., 2020). In Table 4 the six different types of errors (plus no responses) that *sunflower* is able to capture are presented.

**Table 4:** Types of errors that *sunflower* considers regarding speech production.

Error type	Description	Example
No response	No attempt to produce a response.	
Phonemic	Nonword phonologically related to the target word. It contains at least 50% of the phonemes of the target word.	*Tagle* for *Table*
Neologism	Nonword phonologically related to the target word. It contains less than 50% of the phonemes of the target word.	*Timos* for *Table*
Unrelated	Real word that is not related semantically or phonologically to the target word.	*Sneaker* for *Table*
Formal	Real word phonologically related to the target word. It either starts with the same phoneme/letter or contains at least 50% of the phonemes/letters with the target word.	*Truck* for *Table*
Semantic	Real word that is semantically related to the target word.	*Chair* for *Table*
Mixed	Real word that is both phonologically and semantically related to the target word, meeting the criteria for both errors.	*Furnace* for *Furniture*

A response is considered phonologically related if it shares at least 50% of the phonemes with the target word (i.e., *sunflower*’s p_shared_char index ≥ 0.5). A response is deemed semantically related when the semantic similarity, measured as cosine similarity (i.e., cosine_similarity index), is equal to or greater than the cosine limit value (cosine_limit_value parameter) defined in the classify_errors() function (see description of Step 3: Classify Errors).

Before proceeding with the error classification, it is essential to provide *sunflower* with a set of metrics that enable accurate classification. This process involves several stages: (1) to verify whether the responses in the dataframe are real words by means of a lexicality check; (2) to compute formal similarity metrics between the target words and the responses (as done in Step 2: Compute Formal Similarity Measures); and (3) to assess the semantic similarity between the target words and responses. While this process is straightforward when executed in a pipeline manner, as shown in the snippet of code below, some preliminary setup is required. Specifically, we need to load a word2vec model to enable the correct operation of the get_semantic_similarity() function. This can be done using the following line of code before proceeding to run the larger code snippet below:

(6)
(m_w2v = word2vec::read.word2vec(file = file.choose(), normalize = F)

This will lead to a pop-up window on the screen so the user can search for the binary (bin) file containing the model and then load it into the R environment. The one we use, called sbw_vectors.bin, can be found in the dependency-bundle zip allocated in the OSF mirror for *sunflower* (see text footnote 4). Alternatively, it is possible to modify the file parameter to read the binary file directly from a specified route (e.g., file = “models/sbw_vectors.bin”). At this point, we can proceed to obtain the indexes of interest using the initial dataframe obtained in the Step 2, in order to have one response per attempt (df_long_format_w_attempts):

(7)
df_w_all_checks_and_indexes = df_long_format_w_attempts %>%
          get_formal_similarity(item_col = "item",
               response_col = "response",
               attempt_col = "attempt",
               group_cols = c("ID", "item_ID")) %>%
          check_lexicality(item_col = "item",
               response_col = "response",
               criterion = "database") %>%
          get_semantic_similarity(item_col = "item",
               response_col = "response",
               model = m_w2v)

For all the functions in the code above, get_formal_similarity(), check_lexicality(), and get_semantic_similarity(), the parameters item_col and response_col must be used as specified in the previous steps. With regard to the get_formal_similarity() function, we set the parameters in the same way as we did in Step 2: Compute Formal Similarity Measures. However, note that in this case we used the orthographic transcriptions of the items and responses rather than their phonemic transcriptions (i.e., the “item” and “response” columns). In this step, we also introduced two new functions: check_lexicality() and get_semantic_similarity().

The check_lexicality() function allows us to verify whether the responses provided by an individual correspond to real Spanish words or not. This verification process requires a reliable reference source for comparison. Depending on the specified criterion, we have two options: (1) the “dictionary” criterion works by checking if a response is a word present in a pre-loaded wordlist of the *Real Academia de la Lengua Española* (RAE) dictionary (the one made available by Dueñas-Lerín, 2024, version 2024-05-22)^8^ and thus categorizing the response as lexical or non-lexical; (2) the “database” criterion works by checking if the response is a word registered in the Spanish linguistic database *BuscaPalabras* (BPal; Davis and Perea, 2005), and when it is available there, comparing its frequency (the *LOG10_FRQ* from BPal) to that of the item that is the target word being assessed. Both methods provide a systematic way of determining the lexicality of responses, which is crucial for accurate error classification in language production tasks. The choice between these criteria depends on the specific requirements of the research or clinical application. In the former case, a response is considered lexical when it is present in the wordlist. In the latter case, the lexicality of the response is determined by two factors: it must be available in the database and have a higher frequency than the item in question. In any other scenario, it is classified as a non-lexical production. This function creates a column called lexicality, which indicates whether the response is lexical (1) or not (0).

The get_semantic_similarity() function makes it possible to measure the semantic similarity between the target and a response when the latter is a word. This function relies on a natural language processing technique, word2vec, a two-layer neural network model developed by researchers at Google (see Mikolov et al., 2013)^9^ that takes a raw text corpus as input and generates word representations (embeddings) as vectors in a multidimensional space. word2vecthen learns the semantic and syntactic relations of the words within the corpus; this approach has been shown to be effective across diverse semantic tasks (see Kumar, 2021 for a comprehensive review) and its ability to consider the semantic similarity of words is helpful for error categorization, as demonstrated in other studies (Fergadiotis et al., 2016). Models of these kinds determine the relationship by calculating the cosine similarity between the representations of the vectors of two given words, and the more proportional the vectors of the two, the higher cosine similarity and the more semantically similar they are. Neither we nor users are required to build one of these models; rather, we can load one of the preexisting ones available online. This is what we did with the previous line of code used to run the read.word2vec() function from the word2vec package. In our case, we used the vectors contained in the Spanish Billion Word Corpus and Embeddings (see Cardellino, 2016, for further details),^10^ which was called by defining the model parameter as m_w2v with the get_semantic_similarity() function; note that *this model’s name is not written within quotations* as it is not the name of a column, but rather an independent object loaded in R.

With the get_semantic_similarity() function *sunflower* generates a cosine_similarity column that contains the cosine similarity between the vectors of the words in question, in our case the ones provided in the target_col and response_col parameters. Cosine similarity measures might be contained within a [−1,1] interval, but the model we employ uses a [0,1] interval to represent semantic relationships (as in the model used by Salem et al., 2023b), where 0 indicates no semantic association and values near to 1 indicate high semantic similarity values. The user must be aware of this in case they are using a different word2vec model, as they would hence need to adjust certain parameters, such as the cosine_limit_value in the function presented in the following code snippet.

Once we have loaded the word2vec model and obtained the pertinent measures by running a check with the check_lexicality(), get_formal_similarity() and get_semantic_similarity() functions, we already have a dataframe such as df_w_all_checks_and_indexes that is optimal in terms of allowing us to classify the errors.

We note that we have developed two classification functions tailored to work with datasets containing RAs or individual responses. The first function, classify_errors(), allows for error classification while optionally considering RAs, offering greater flexibility through a larger set of configurable parameters. The second function, classify_errors_regular(), is specifically designed for individual responses, disregarding RAs entirely, and resulting in a simpler setup with fewer parameters. Starting with the former, classify_errors(), error classification can be achieved by running the following code snippet:

(8)
dataframe_w_classified_errors = df_w_all_checks_and_indexes %>%
     classify_errors(access_col = "accessed",
          RA_col = "RA",
          response_col = "response",
          item_col = "item",
          also_classify_RAs = TRUE,
          cosine_limit_value = 0.46)

When the access_col parameter refers to whether the produced response was annotated as successful or not. This might be done manually by the user or automatically by adding another line to the code, such as a conditional statement, returning 1 if the item and response are equal, or 0 if they are not (see the third line in the following code snippet). The parameter RA_col indicates whether or not the production is a RA such as a CdA (RA [1], single response [0]); in our case this was the RA column obtained in the previous steps. The item_col and response_col are the same as those used in the previous steps. The parameter also_classify_RAs here is set as TRUE (or T) to allow the function to work by considering the classification of productions within the RAs (when value in “RA” is 1) as any of the 6 possible error categories (nonword, neologism, formal, unrelated, mixed, semantic). However, it can be set as FALSE (or F) so the function will leave the productions within the RAs (when value in the “RA” is 0) unclassified.

The classify_errors() function also uses a parameter called cosine_limit_value. This parameter is used to classify items and responses as semantically similar or not, based on whether the cosine similarity value provided by the get_semantic_similarity() function (i.e., w2v_cos) exceeds a specified threshold. As mentioned earlier, in our model, the w2v_cos values are bound within the range [0, 1] (although other models may define the bounds within the interval [−1, 1], and in such cases the cosine_limit_value parameter should be adjusted accordingly.). In our package, this parameter is set to 0.46 by default, although it can be adjusted to make the classification process more or less stringent. In their study, for example, Salem et al. (2023b) used the slightly higher threshold of 0.55. While this value may appear stricter, it aligns with the classification criteria used in their research. Note that Salem et al. (2023b) and our study use different stimulus–response pair samples, different languages (theirs being English, ours Spanish), and the models were trained on distinct datasets. As a result, the cosine similarity values—while always bound within [0, 1]—may differ in how they represent word representations and their relationships. Naturally, this may lead to differences in determining whether pairs are semantically related. Although there is no consensus on this matter, setting the threshold to around 0.50 would be a safe practice toward reliably detecting relationships. Nonetheless, we remind users that it can and should be adjusted to meet their specific criteria. Additionally, it is important to note that the get_semantic_similarity() function returns an NA value for the cosine similarity, w2v_cos, when either the item or the response to be considered is NA.

**Figure 8:**
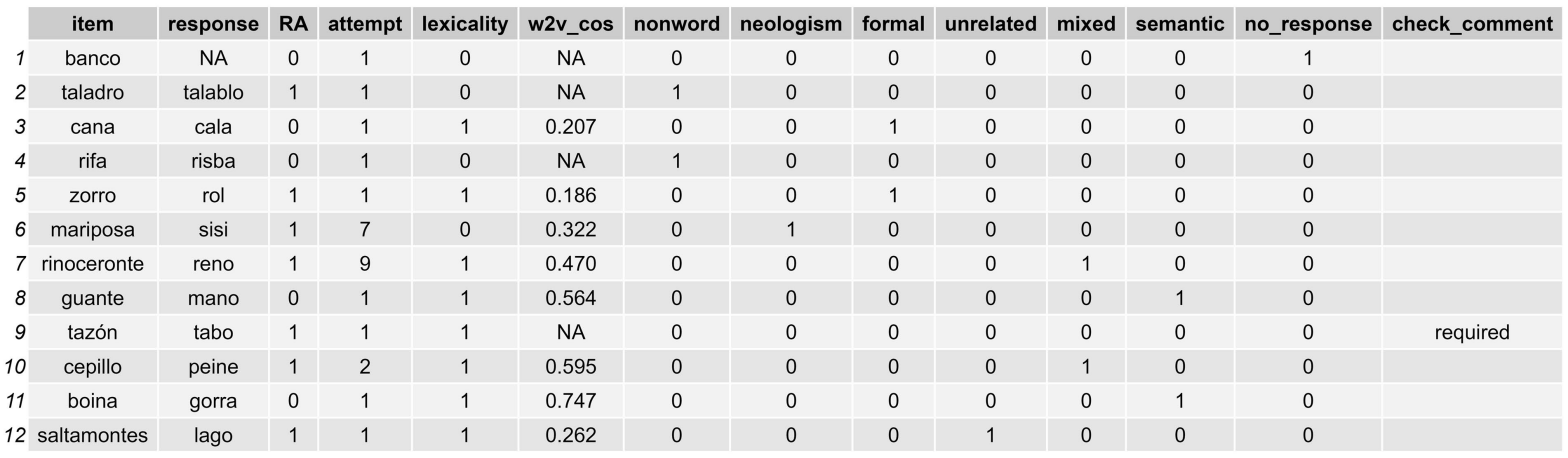
Dataframe displaying the errors classified by using the *sunflower* package.

Finally, we obtain a final dataframe, which here we have named df_w_classified_errors, with a series of columns displaying the desired error classification conducted with *sunflower*: nonword, neologism, formal, unrelated, mixed, and semantic, but also no responses. We also obtain a check_comment column that flags responses as “required” in case they need to be explicitly addressed manually when no possible classification can be made (i.e., when the value is zero in all the nonword, neologism, formal, unrelated, mixed, and semantic columns) or, on the other hand, when a multiple classification is made (when the response has been classified as an error in more than one column). Additionally, it provides the comment “is only considered as RA” for responses contained in RA instances when the also_classify_RAs parameter is set as FALSE. Otherwise, being set as TRUE, it remains empty. The classification obtained here is shown in Figure 8.

As a side note, users can achieve the same result by chaining multiple functions instead of generating new data frames at each step. This approach is particularly suited for those with advanced expertise in R or familiarity with the package. In the following code snippet, the process begins with the initial data frame (IGC_sample) and uses a stacked approach to produce the same df_w_classified_errors (but _stacked in this case).

(9)
df_w_classified_errors_stacked = IGC_sample %>%
     separate_responses(col_name = "response",
          separate_with = ", ") %>%
     get_attempts(drop_blank_spaces = TRUE) %>%
     get_formal_similarity(item_col = "item",
          response_col = "response",
          attempt_col = "attempt",
          group_cols = c("ID", "item_ID")) %>%
     check_lexicality(item_col = "item",
          response_col = "response",
          criterion = "database") %>%
     get_semantic_similarity(item_col = "item",
          response_col = "response",
          model = m_w2v) %>%
     classify_errors(access_col = "accessed",
          RA_col = "RA",
          response_col = "response",
          item_col = "item",
          also_classify_RAs = TRUE,
          cosine_limit_value = 0.46)

As mentioned above, we also developed a second version of this function for cases where the user wishes to classify single errors (i.e., without RAs) in a more traditionally organized dataframe. This is the classify_errors_regular() which allows us to work without considering the RA_col and also_classify_RAs parameters of the other function. This simplifies the code by relying on fewer parameters. In the following code snippet, we provide a hypothetical case in which we work with no RAs after filtering them out of our dataframe using the *dplyr* package:

(10)
df_w_classified_errors_noRAs = df_w_all_checks_and_indexes %>%
     dplyr::filter(RA == 0) %>%
     dplyr::mutate(accessed = ifelse(item == response, 1, 0)) %>%
     classify_errors_regular(access_col = "accessed",
          response_col = "response",
          item_col = "item",
          cosine_limit_value = 0.46)

Thus far, we have showcased several features of the *sunflower* package, from data wrangling of multiple responses to error classification. Users can find additional example code in the vignettes available on GitHub at https://github.com/ismaelgutier/sunflower. A detailed guided tutorial is also accessible and can be downloaded directly from our OSF repository via the following link: https://osf.io/urz4y.

## 5 Discussion

In this paper we present *sunflower*, an R package that allows for the handling of large dataframes not only to obtain various measures related to the quality of productions from a patient or participant transcription, but also to serve as an automatic classifier of the errors made by them.

The value of this tool lies in the handling of dataframes that contain RAs, such as the CdAs typically produced by patients with aphasia, especially conduction aphasia, by children with language disabilities, and by persons who stutter. It allows for the study of the formal qualities of these productions by obtaining multiple complex measures based on algorithms that are more informative than those that, with significant effort, can be calculated manually by clinicians or researchers. Additionally, it enables the exploration in depth of the quality of productions at a fine-grained level—phonemes when working with phonemic transcriptions or letters when using orthographic transcriptions.

Likewise, the present software is able to test the lexicality of productions on the basis of preexisting Spanish wordlists and databases, and can produce semantic similarity measures by using pretrained AI models, namely those based on the word2vec technique, for the subsequent classification of production errors following established criteria in the field.

Previous studies have shown how tools of this kind can be used to classify errors and how such procedures are indeed reliable (e.g., Fergadiotis et al., 2016; Salem et al., 2023a; Schnur and Lei, 2022). Unfortunately, they do not publicly share the tools they develop and use so that others can conduct additional work using them. Our package is designed not only to provide this, but also to offer the possibility of conducting a formal analysis of words. It is developed in Spanish, a language that has not been the focus of a great deal of work of this kind. It is designed so that both researchers and clinicians (neuropsychologists and speech therapists), who often do not have extensive programming knowledge, can access a comprehensive tool for studying language production errors. This tool also allows them to perform statistical analyses based on the outputs obtained.

The limitations are clear: in order to apply some of its functions, the package relies on databases, dictionaries, and pre-trained models, which may affect the quality of its performance. However, users are not limited to using the dependencies we cite in the text or in our own repositories and can, for example, load their own trained models. In all cases, the outputs should be supervised by an expert, especially during the final stage of the process, where the word2vec model and its ability to represent semantics come into play. While earlier stages rely primarily on algorithms that work with formal representations that leave little room for ambiguity, word2vec focuses on learning the relationships between words within the corpus, mapping them to multiple internal dimensions of the model. Although the word2vec technique has proved to be effective in various semantic tasks (Kumar, 2021), the material on which these models are trained means that word representations are not always identical, and variations may arise when comparing them to human logic and reasoning, particularly when interpreting relationships between specific word pairs (e.g., stimulus–response). These may not always perfectly match human classification criteria. Therefore, careful supervision at this stage is crucial to ensure that results remain consistent and of high quality. Like any flower, *sunflower* will bloom best when provided with quality soil and when it receives careful attention from a gardener.

In sum, we offer a tool that is accessible to everyone, allowing it to be used for tasks such as the focused study of production quality, the effect of treatments on error production, and the better diagnosis and study of various conditions, including aphasia, apraxia, stuttering and developmental speech sound disorders.

Future possible directions of this project include extending the development of the package to other languages. In its present form the *sunflower* package is designed to be used with Spanish stimuli, yet adapting it to any other language would be feasible. Indeed, for the initial steps related to data wrangling (Step 1) and the computation of formal quality indexes (Step 2), there is no need to change anything. Regarding Step 3, which is conceived to include a lexicality check procedure, as well as the computation of the abovementioned formal quality indexes and a semantic similarity index, small changes to the code and dependencies could be made to allow users to work with data from different languages. Adaptation work here would involve finding equivalent databases and corpus- or dictionary-based wordlists, as well as to set use other word2vec models to allow the lexicality check and the semantic similarity cosine computation.

We hope that *sunflower* proves to be a useful resource, and that it lightens the workload of coding errors for other researchers as it has for us.

### Software basic requirements

*sunflower* is freely available at GitHub (https://github.com/ismaelgutier/sunflower) and its mirror OSF (https://osf.io/akuxv/); operating system(s): *Windows*; programming language: *R*; dependencies: *tidyr*, *dplyr*, *tidyverse*, *reshape2*, *stringdist*, *stringr*, *PTXQC*, *tibble*, *tictoc*, *magrittr*, *purrr*, *rlang*, *stats*, *word2vec*. The installation packages for all the required software are available at the *sunflower* repositories. A dependency bundle with some “additional” source files to work (in Step 3: Classify Errors) is provided at OSF. Users do not need to download the required software individually. The *sunflower* home page also provides users with examples for reference. There are no restrictions on non-academic use; in fact, such use is encouraged.

### Registration

The registration of *sunflower* was made in OSF (https://osf.io/bw4az) to clarify its motivation in advance and to assist in preserving the essence of the project throughout its development and maintenance.

A portion of Gutiérrez-Cordero and García-Orza’s (in preparation) data was analyzed in this study as an example. Other sample data made available to test the functions of *sunflower* are provided with the package, and other supplementary data can be accessed through the GitHub repository (https://github.com/ismaelgutier/sunflower) or directly through the mirror repository at OSF (https://osf.io/akuxv/).

## Data Availability

The registration of sunflower was made in OSF (https://osf.io/bw4az) to clarify its motivation in advance and to assist in preserving the essence of the project throughout its development and maintenance. A portion of Gutiérrez-Cordero and García-Orza’s (in preparation) data was analyzed in this study as an example. Other sample data made available to test the functions of sunflower are provided with the package, and other supplementary data can be accessed through the GitHub repository (https://github.com/ismaelgutier/sunflower) or directly through the mirror repository at OSF (https://osf.io/akuxv/).

## Footnotes

^1^ http://www.R-project.org/

^2^ https://posit.co/products/open-source/rstudio/

^3^ https://github.com/ismaelgutier/sunflower

^4^ https://osf.io/akuxv/

^5^*sunflower* uses the pipe operator (% > %) from the *tidyverse*, so it can be used to enhance any workflow along with other functions integrated into its framework, such as those from *dplyr* for data wrangling, *readr* for data reading, and *ggplot2* for data visualization.

^6^To avoid any subsequent problems, we suggest not changing the name of the resulting columns as the functions work with some columns as internally defined. If they wish, users can rename these columns after computing all the metrics and classifying the errors.

^7^A way to work with phonemic transcriptions is to write them directly using IPA symbols in an Excel file (or some other file type) or deriving a transcription from a previous orthographic one with the added support of audio data for reliability. Given the regularity and shallowness of Spanish, this practice is quite easy to achieve, but is still rather time consuming. A further alternative is to automatically obtain these phonemic transcriptions by using a transcriber, such as the *IAR-transcriber* library (Arias Rodríguez, 2021; https://pypi.org/project/iar-transcriber/) in *Python* (Van Rossum and Drake, 2009), on the previous orthographic ones, and then supervise the outcome. This procedure has been demonstrated to be reliable in the case of a Castilian Spanish patient with phonological impairments as described in another study of ours (Gutiérrez-Cordero and García-Orza, in preparation). In that study we used some of the in-development-stage functions of *sunflower* to work with IPA phonemic transcriptions after cleaning them of any possible superscripts and diacritics. Note that these are considered as independent elements in the strings to work with, and some of them are not recognized by R, so this procedure was conducted to keep these transcriptions as simple as possible so that R could successfully handle them. We suggest that interested users conduct automatic transcriptions of this kind once the dataframe is in long-format (as we did with the sunflower::IGC_long_sample dataframe to obtain the sunflower::IGC_long_phon_sample) in case they want to compute the formal metrics of the phonemic transcriptions to ease the procedure of revising and cleaning them before computing such metrics. Furthermore, we recommend that users supervise the outputs of transcriptions to ensure their reliability, and hence that of subsequent computations.

^8^ https://github.com/JorgeDuenasLerin/diccionario-espanol-txt

^9^ https://code.google.com/archive/p/word2vec/

^10^ https://crscardellino.github.io/SBWCE/
